# The association of early antibiotic exposure with subsequent development of late-onset sepsis in preterm infants: a systematic review and meta-analysis studies

**DOI:** 10.1186/s12245-025-00869-5

**Published:** 2025-04-18

**Authors:** YF Shamseldin, Heba Khaled, Muhammed Abdiwahab, Maha K. Abu Radwan, Abdalfattah Sabra, Mona Mohammed, Sarah EL-Sayegh, Dina Abdel Rasoul Helal, Mahmoud E. Kamal, Ahmed Hassan, Ahmed Azzam

**Affiliations:** 1 Department of Clinical Pharmacy, College of Pharmaceutical Sciences and Drug Manufacturing, Misr University for Science and Technology, Giza, Egypt; 2 Department of Biochemistry, Faculty of Pharmacy, Cairo University, Cairo, Egypt; 3 Tanta University Hospitals, Tanta, Egypt; 4 Princess Muna College of Nursing, Mutha University, Mutha, Jordan; 5 Al-Zagazig University AL Zagazig, Zagazig, Egypt; 6 Pediatric resident Doctor at National Nutrition Institute, Cairo, Egypt; 7 Department of Clinical and Chemical Pathology, Faculty of Medicine, Cairo University, Cairo, Egypt; 8 Department of Pediatrics, Faculty of Medicine, Mansoura University, Mansoura, Egypt; 9 Clinical Pharmacy and Pharmacy Practice Department, Faculty of Pharmacy, Egyptian Russian University, Cairo, Egypt; 10 Department of Clinical Pharmacy, Faculty of Pharmacy, University of Sadat City, Sadat City, Menoufia 32897 Egypt; 11 Department of Microbiology and Immunology, Faculty of Pharmacy, Helwan University, Cairo, Egypt

**Keywords:** Preterm infants, Early antibiotic exposure, Late-onset sepsis, Meta-analysis

## Abstract

**Background:**

Early antibiotic exposure in preterm infants may disrupt gut microbiome development, affecting health. However, its link to late-onset sepsis (LOS) remains unclear. This meta-analysis aims to clarify the association while addressing confounding bias.

**Methods:**

This systematic review and meta-analysis, conducted per PRISMA guidelines, utilized PubMed, Scopus, Google Scholar, and Web of Science for comprehensive literature retrieval. Studies comparing preterm infants with sterile blood cultures who received early antibiotics (short or prolonged) to those without, using LOS as the primary outcome, were included. Comparisons between short- and prolonged-course antibiotics were also considered. Only studies with adjusted analyses for confounders were considered. Adjusted odds ratios (aOR) were meta-analyzed, and the prediction interval (PI) was calculated using R software.

**Results:**

Ten studies met the eligibility criteria, comprising a total sample size of 55,089 preterm infants. Among these, nine studies included 33,549 preterm infants and compared prolonged antibiotic exposure to short exposure. Prolonged exposure was not significantly associated with LOS (pooled aOR = 1.2, 95% CI 0.99–1.46, *P* = 0.066, PI = 0.66 to 2.19, I² = 67%). Limiting the analysis to five studies with sample sizes over 1,000 reduced heterogeneity (I² = 30%) and provided a more precise confidence interval (pooled aOR = 1.03, 95% CI 0.91–1.15). Four studies, involving 41,938 preterm infants, examined preterm infants exposed to prolonged antibiotics versus those not exposed and found no significant association (aOR = 0.91, 95% CI 0.82–1.02, *P* = 0.1, PI = 0.72 to 1.16, I² = 0). All four studies had sample sizes exceeding 1,000. Additionally, these studies compared preterm infants with short antibiotic exposure to non-exposure, revealing a slightly lower risk of LOS (aOR = 0.87, 95% CI 0.77–0.98, *P* = 0.024, I² = 0) and a PI of 0.76 to 1.14.

**Conclusions:**

Our findings indicate that prolonged early antibiotic exposure in preterm infants with sterile cultures does not significantly increase the risk of LOS compared to no antibiotic exposure. Interestingly, a shorter duration of antibiotic exposure might be associated with a slightly lower risk of LOS.

**Supplementary Information:**

The online version contains supplementary material available at 10.1186/s12245-025-00869-5.

## Background

Neonatal sepsis is one of the primary causes of mortality at the neonatal intensive care unit (NICU) [1]. In addition, it is the leading driver of antibiotic prescriptions in NICU [2]. It typically presents modest and nonspecific signs overlapping with many clinical conditions, for instance prematurity-related events. Therefore, clinicians often extend empirical antibiotic therapy even in the absence of positive blood cultures or clinical signs [3–5]. Antibiotics are initially administered to prevent delayed sepsis diagnosis, while their prolonged use aims to reduce the risk of relapse or undertreatment. A worldwide investigation involving preterm infants in 84 NICUs across 29 countries showed that 92% of infants received antibiotics [4].

This unnecessary antibiotic treatment may result in several neonatal adverse outcomes, especially in early life. Previous studies indicate that antibiotic exposure in preterm infants with sterile cultures increases the risk of experiencing adverse events such as necrotizing enterocolitis (NEC) [6], late-onset sepsis (LOS) [7–9], and mortality [8, 10]. However, the evidence concerning LOS remains inconclusive. Some studies suggest an increased risk of LOS [7–9], however, these studies have smaller sample sizes, which could potentially lead to an overestimation of the odds ratio due to the small sample size. Others find no significant association [3, 11–13]. Moreover, a recent case-control study showed that antibiotic exposure before the onset of LOS was associated with decreased odds of gram-positive LOS [14]. Similarly, a prior study utilizing preterm pigs as a model demonstrated that oral administration of broad-spectrum antibiotics to preterm infants delayed gut bacterial colonization, improved blood neutrophil maturation, and provided protection against bacteremia [15].

In this study, our objective was to identify, critically assess, and synthesize evidence from studies that investigate the association of early antibiotic short or prolonged exposure in preterm infants with negative cultures with the subsequent risk of LOS. To mitigate the potential for confounding, we selectively included studies that adjusted for confounders. Furthermore, we stratified the results based on sample size to minimize the overestimated risk in studies with a small sample size.

## Methods

### Search strategy

We conducted a thorough literature search from inception to June 1, 2024, using PubMed, Scopus, Google Scholar, and Web of Science databases. The detailed search strategy is provided in Table S1. Additionally, the reference lists of all included studies were systematically screened to identify any additional eligible articles. This systematic review followed the Preferred Reporting Items for Systematic Reviews and Meta-Analyses (PRISMA) guidelines. Table S2 includes the PRISMA checklist outlining the items covered in this systematic review’s reporting.

### Eligibility criteria

#### Inclusion criteria (PICO Format)

The inclusion criteria were structured using the PICO format as follows:

Population: Preterm infants with sterile blood cultures.

Intervention: Exposure to antibiotics for a specified duration, either a prolonged or short period of exposure.

Comparison: Preterm infants who received antibiotic therapy (prolonged or short course) versus those who did not. Additionally, studies comparing short-course vs. prolonged antibiotic therapy were included.

Outcome: blood culture-confirmed LOS cases.

Additional Criteria: Studies included adjusted analyses to account for potential confounding factors.

#### Exclusion criteria

Preterm infants who developed culture-proven sepsis, case reports, letters, reviews, not peer-reviewed publications and editorials.

### Study selection

We initiated the search by entering relevant keywords into the selected databases, creating individual libraries for each database. Duplicates were removed using Zotero 6.0. We then proceeded with the step-by-step selection of eligible articles. Initially, we excluded articles based on their titles, eliminating those deemed completely irrelevant. If there was any doubt, we retained the article for further evaluation in subsequent steps. The remaining articles were assessed for eligibility based on their abstracts and full texts. Additionally, we reviewed the reference lists of the eligible articles to identify any potential additional eligible studies. Six reviewers independently selected the relevant articles based on the previously mentioned inclusion and exclusion criteria.

### Definitions

LOS was defined as the isolation of pathogenic bacteria from blood and/or cerebrospinal fluid cultures, or fungi from blood cultures, occurring more than 72 h after birth [16]. The definition of early empirical antibiotic administration varied across studies; however, for the purposes of this study, it was considered as antibiotics administered within the first week after birth encompassing both short-course and prolonged exposure. There is no consensus definition for prolonged and short antibiotic exposure antibiotics, so we report the specific definitions used by each study’s authors.

### Data extraction

Two reviewers independently extracted the necessary data from each included study. Two additional reviewers then cross-checked the extracted data to ensure accuracy and consistency.

### **Risk of bias assessment**

To assess the risk of bias of the included studies, we employed the Newcastle Ottawa Scale (NOS) [17]. This scale evaluates studies in three key areas: selection, comparability, and outcome. The checklist items are presented in Table S3. The NOS scoring system has a maximum of 9 points, with studies receiving a maximum of one point for each criterion within the selection and outcome domains. In the comparability domain, a maximum of two points can be awarded. Each eligible article was independently assessed by three reviewers, and any scoring discrepancies were resolved through discussion and consensus among all authors.

### Data synthesis

We reported the results as pooled adjusted odds ratios (aOR) with 95% confidence intervals (CI). We conducted a subgroup analysis of studies with over 1000 participants to mitigate the overestimation of ORs [18] and minimize the risk of publication bias in smaller studies. The pooled estimates were calculated under the random-effects model, which accounts for variability both within and between studies. Heterogeneity between the studies was assessed using I-squared. In addition, we reported the prediction interval (PI), which indicates the range within which the true effect size is expected to fall for 95% of all populations [18]. Publication bias was assessed using a funnel plot and Egger’s regression test. All statistical analyses were performed using the R programming language (version 4.0.2). Specifically, the meta-analysis was conducted utilizing the ‘meta’ and ‘metafor’ packages.

## Results

### Characteristics of eligible articles

**Fig. 1:**
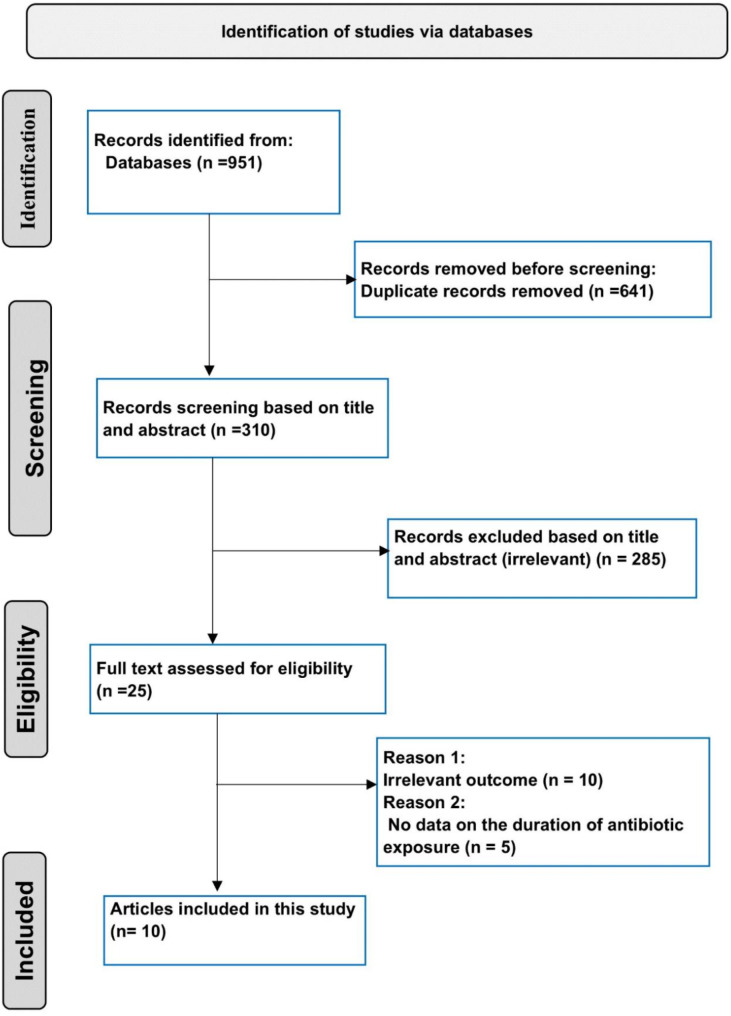
PRISMA flow diagram of study selection process

This systematic review included ten original research studies with a total of 55,089 preterm infants [3, 7–9, 11–13, 19–21] as shown in Fig. 1. The characteristics of eligible articles are displayed in Table 1. In four studies, the sample sizes were relatively small, with less than 1000 infants [7–9, 19]. Conversely, six studies had larger sample sizes, with more than 1000 infants [3, 11–13, 20, 21]. Among these, two large studies included over 10,000 infants [13, 21]. Eight studies were conducted across multiple centers [3, 7, 11–13, 19–21]. In contrast, two studies were conducted at a single center [8, 9]. All studies followed a retrospective observational study design, except for those that utilized a prospective observational study design [8, 12]. Antibiotics were started early in all the studies included. The definition of prolonged exposure to antibiotics varied across the ten studies: lasting for ≥ 5 days in five studies [3, 7, 9, 20, 21]; for ≥ 4 days in four studies [8, 11, 13, 19]; and for > 3 days in one study [12]. All the studies employed multivariable logistic regression for the adjustment of the confounders. The overall risk of bias assessment in Table 1 was low, although some studies had a higher risk (lower scores, such as 7) due to small sample sizes and inadequate adjustment for confounders [7, 19].

Other characteristics include setting/country, study design, birth years of cohort, total numbers of participants, total number of preterm infants with either prolonged or short early exposure to antibiotics. Others without exposure to antibiotics (0 days), and Late-Onset Sepsis Cases by Duration of Exposure; all are included in table S4.

**Table 1:** Characteristics of the included studies

Last name of the first author (publication year)	Total number of preterm infants	# Centers	Gestational age or weight	Cutoff of prolonged antibiotic exposure (Days)	Adjustment methods	Adjusted factors	NOS (out of 9)
Kuppala (2011) [7]	305	Multicenter	< 32 weeks	≥ 5	Multivariable logistic regression	**Maternal Factors** PROM **Infant Factors** Birth weight, GA, Race **Clinical Factors** High-frequency ventilation Received breast milk and Apgar score at 5 min	**8**
Cotten (2009) [3]	5693	Multicenter	ELBW	≥ 5	Multivariable logistic regression	**Maternal Factors** prenatal steroid use, prenatal antibiotic use, hypertension, PROM, hemorrhage **Infant Factors** race, gender, GA, SMA, multiple births **Clinical Factors** 5-minute Apgar score, center, mechanical ventilation, early initiation of enteral feeds	**9**
Ting (2019) [13]	14,207	Multicenter	VLBW	≥ 4	Multivariable logistic regression	**Maternal factors** PROM **Infant factors** GA, multiple births **Clinical factors** SNAP-II scores, extensive CPR, surfactant use, mechanical ventilation, inotropes and pneumothorax treated with chest tube	**9**
Greenberg (2019) [20]	5730	Multicenter	< 28 weeks	≥ 5	Multivariable logistic regression	**Maternal factors** Black race, Multiple birth, PROM > 24 h, Receipt of antenatal steroids, Perinatal antibiotic therapy, Hypertension, Antepartum hemorrhage, Cesarean section **Infant factors** GA, SGA, Sex **Clinical factors** 5-min Apgar score < 5, Birth year, Center, Mechanical ventilation	**9**
Dierikx (2022) [12]	1259	Multicenter	< 30 weeks	> 3	Multivariable logistic regression	**Maternal factors** Mode of delivery (vaginal, c-section) **Infant factors** Gender, Birth weight percentile, GA **Clinical factors** Center, Days of parenteral feeding, Invasive ventilation support, Inotropic medication use type of enteral feeding and Apgar score 5 min	**9**
Fajardoa (2018) [9]	620	Single center	VLBW	> 5	Multivariable logistic regression	**Maternal factors** Maternal hypertension, Prenatal steroid treatment and Intrapartum antibiotic treatment **Infant factors** SGA, Gender and Multiple births **Clinical factors** SNAP II and Clinical chorioamnionitis	**8**
Yu (2023) [21]	21,540	Multicenter	< 34 weeks	≥ 5	Multivariable logistic regression	**Maternal Factors** Maternal hypertension, Maternal diabetes, Maternal steroid use, Cesarean section **Infant Factors** GA, Male, SGA, 5-minute Apgar score ≤ 7 **Clinical Factors:** Mechanical ventilation, Vasopressor treatment, Breastmilk feeding, Age of enteral feed initiation	**9**
Vatne (2023) [11]	4932	Multicenter	< 32 weeks.	≥ 4	Multivariable logistic regression	**Maternal Factors** Mode of delivery, Antenatal steroids **Infant Factors** GA, Sex, Multiple births, Birth weight, Year of birth, Birth region **Clinical Factors** Antibiotics, Days of mechanical ventilation, CRIB2, Apgar score, Intraventricular hemorrhage	**9**
Shah(2013) [8]	216	Single center	< 28 weeks	≥ 4	Multivariable logistic regression and similar baseline characteristics	**Maternal factors** PIH, APH, Mode of delivery, Maternal antibiotics, Antenatal glucocorticoids **Infant factors** GA, Birth weight, IUGR, gender **Clinical factors** Chorioamnionitis, PROM, CRIB score, Apgar score	**7**
Alsafadi (2018) [19]	587	multicenter	< 34 weeks	≥ 4	Multivariable logistic regression	**Birth weight**,** gestational age**,** gender**,** mode of delivery**,** PROM**,** pregnancy-induced hypertension**,** multiple births**,** asphyxia**,** sepsis laboratory tests**	**7**

**NOS**,** Newcastle Ottawa Scale; PROM**,** Premature Rupture of Membranes; GA**,** Gestational Age; CRIB**,** Clinical Risk Index for Babies; SGA**,** Small for Gestational Age; IUGR**,** Intrauterine Growth Restriction; PIH**,** Pregnancy-Induced Hypertension; APH**,** Antepartum Hemorrhage; Apgar**,** Appearance**,** Pulse**,** Grimace**,** Activity**,** Respiration; SNAP-II**,** Score for Neonatal Acute Physiology-II; SMA**,** Sall for Gestational Age**

### Risk of LOS in preterm infants exposed to prolonged antibiotics vs. Short exposure

**Fig. 2:**
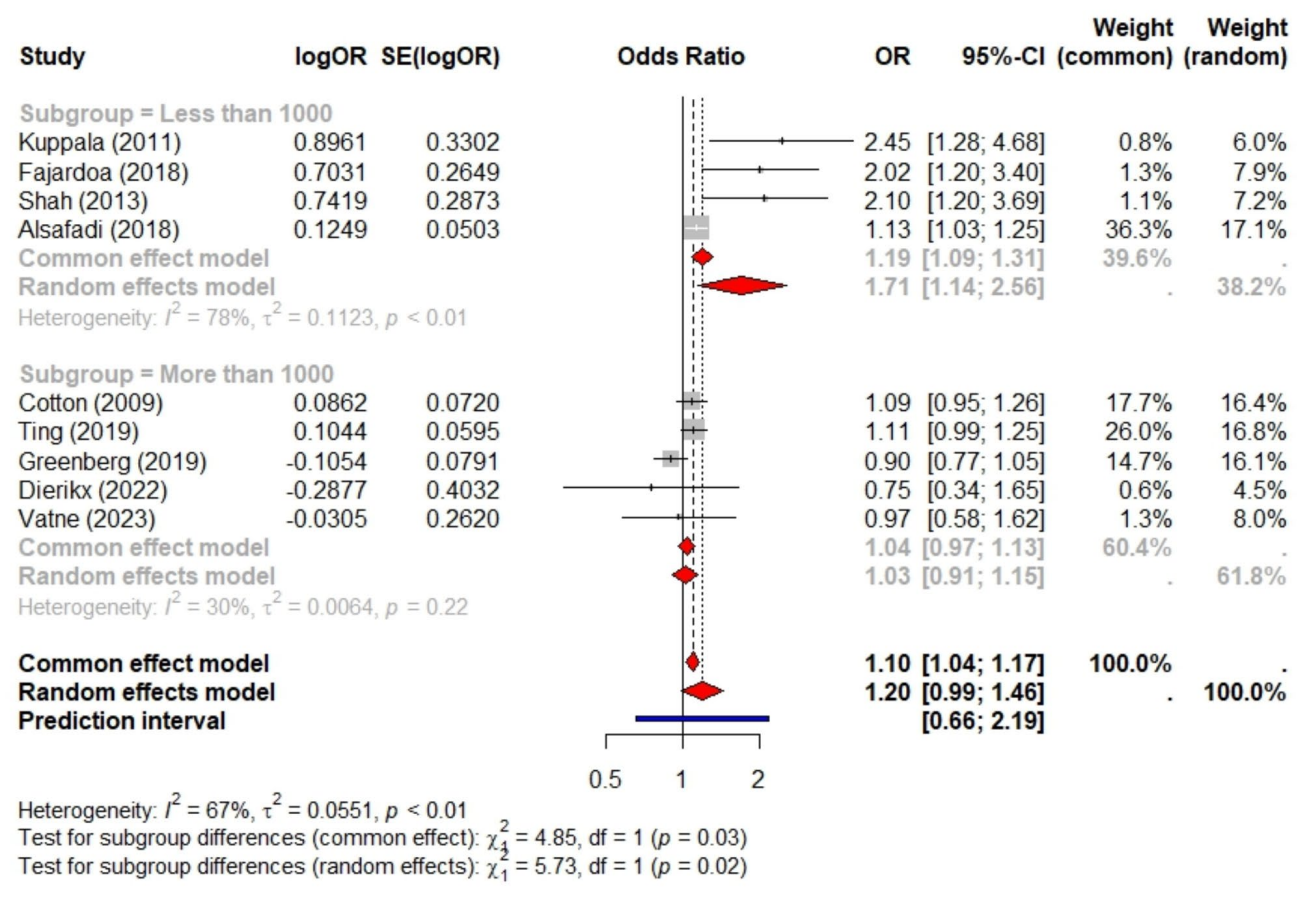
Meta-analysis of the risk of late-onset sepsis (LOS) in preterm infants exposed to prolonged versus short antibiotic courses, stratified by a sample size of 1000. This analysis includes nine studies with a combined total of 33,549 preterm infants

**Fig. 3:**
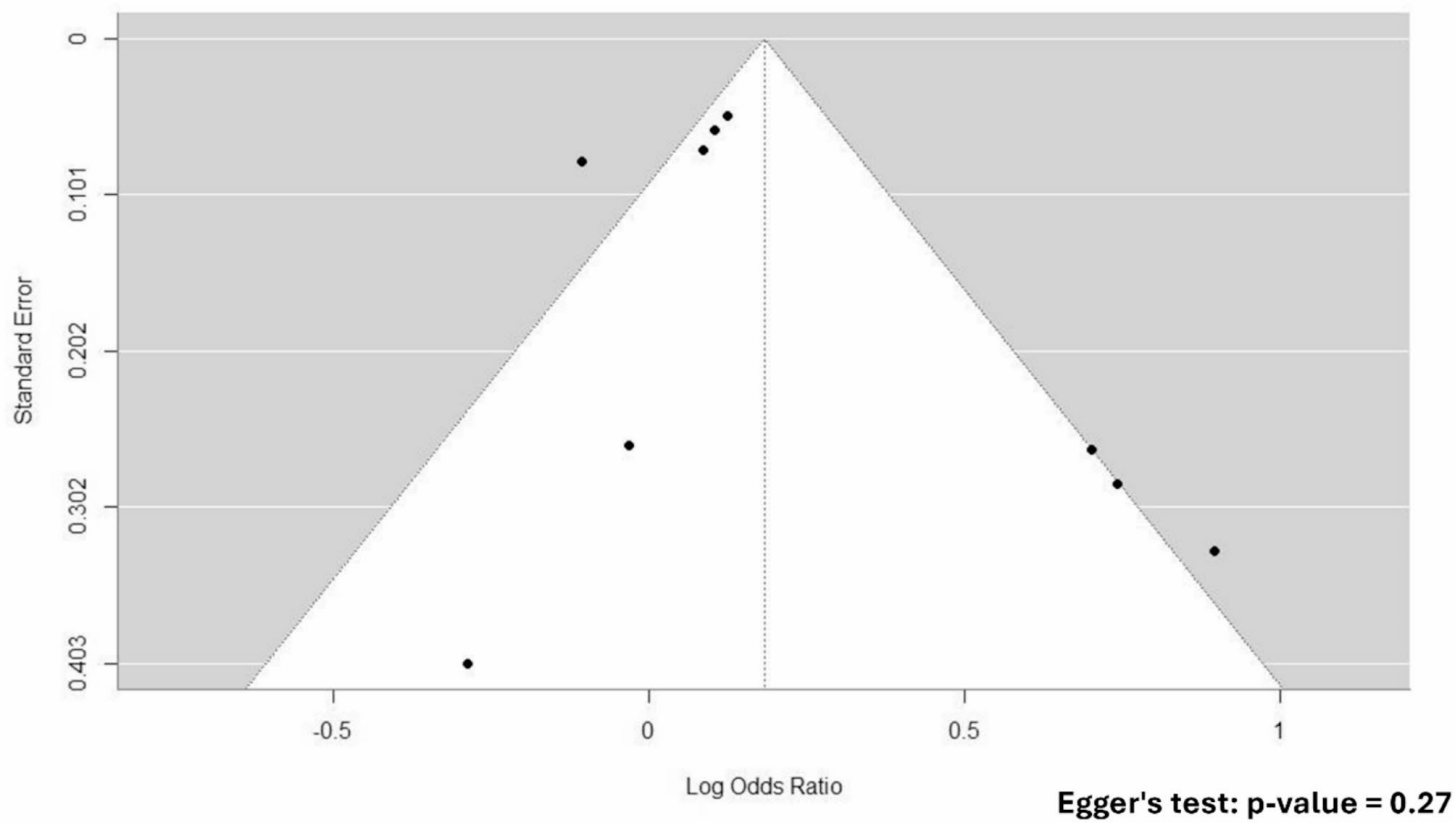
Funnel plot of the risk of late-onset sepsis (LOS) in preterm infants exposed to prolonged versus short antibiotic exposure. Egger’s regression analysis showed no evidence of publication bias (Egger’s test, *p* = 0.27)

Nine studies, involving a total of 33,549 preterm infants, investigated the risk of LOS associated with prolonged antibiotic exposure compared to short exposure (defined as > 5 days vs. <5 days in five studies and ≥ 4 days vs. <4 days in three studies). Prolonged antibiotic exposure was not significantly associated with an increased risk of LOS (pooled aOR = 1.2, 95% CI 0.99–1.46, *P* = 0.066), as illustrated in Fig. 2. The prediction interval was wide, ranging from 0.66 to 2.19. Additionally, substantial heterogeneity was observed among these studies (I² = 67%). Publication bias testing using the funnel plot and Egger’s regression analysis revealed no evidence of publication bias, with an Egger’s test p value of 0.27, as shown in Fig. 3. Restricting the analysis to five studies with sample sizes exceeding 1000 resulted in decreased heterogeneity (I² = 30%, total sample size = 31,821) and a non-significant association between prolonged exposure and LOS, with a narrower confidence interval and greater precision (pooled aOR = 1.03, 95% CI 0.91–1.15), as delineated in Fig. 2.

The subgroup analysis of studies with sample sizes less than 1000 showed a pooled aOR of 1.71 (95% CI 1.14 to 2.56), indicating a significant association between prolonged antibiotic exposure and LOS. This subgroup exhibited substantial heterogeneity, as evidenced by an I² value of 78%.

### Risk of LOS in preterm infants exposed to prolonged antibiotics vs. non-exposed

**Fig. 4:**
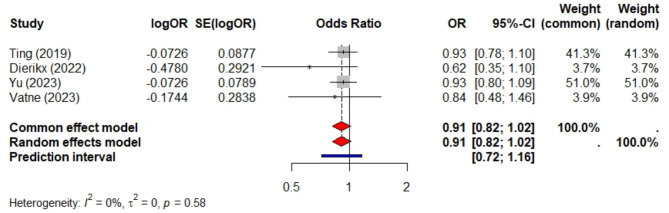
Meta-analysis of the risk of late-onset sepsis in preterm infants exposed to a prolonged course of antibiotics versus non-exposed. This analysis includes four studies with a combined sample size of 41,938 preterm infants

Four studies, encompassing a total sample size of 41,938 preterm infants, reported a pooled aOR of 0.91 (95% CI 0.82–1.02, *p* = 0.1) for the association between prolonged antibiotic exposure and LOS. The PI of 0.72 to 1.16 further supports the lack of a statistically significant effect, as shown in Fig. 4. In all of these studies, sample sizes were over 1000, and the results were homogeneous, as evidenced by the I^2^ of 0.

### Risk of LOS in preterm infants with short antibiotic exposure vs. non-exposure

**Fig. 5:**
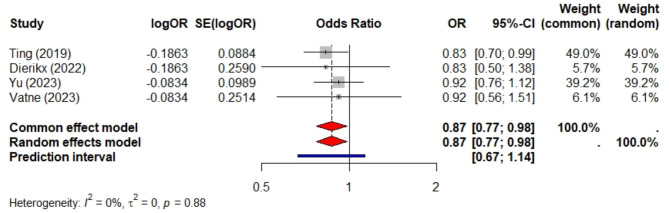
Meta-analysis of late-onset sepsis in preterm infants exposed to a short course of antibiotics versus non-exposed. This analysis includes four studies with a combined sample size of 41,938 preterm infants

Four studies, encompassing a total sample size of 41,938 preterm infants, were meta-analyzed. All of these studies had sample sizes exceeding 1000, and the results were homogeneous, as evidenced by an I² of 0%. Overall, the risk of LOS was slightly lower for preterm babies who were exposed to antibiotics for a short duration compared to those who were not exposed (pooled aOR of 0.87, 95% CI 0.77–0.98, *P* = 0.024) with a slightly wide prediction interval of 0.67 to 1.14 as shown in Fig. 5.

## Discussion

To our knowledge, this Meta-analysis is the first to investigate the risk of subsequent LOS in preterm infants with sterile cultures exposed to varying durations of antibiotics in early life. All the included studies adjust for neonatal, maternal and clinical confounders. Overall, our findings suggest that neither prolonged nor short antibiotic exposure is significantly associated with an increased risk of LOS in preterm infants. However, short antibiotic exposure may be linked to a slightly lower risk.

Antibiotic exposure in preterm infants can disrupt the natural balance of the gut microbiota, leading to a condition known as dysbiosis [22]. This disruption can significantly affect the development and function of the immune system, especially in preterm infants whose immune systems and microbiotas are still maturing [22]. The altered microbiota can impair both innate and adaptive immune responses, reducing the effectiveness of immune defenses against infections [22]. These changes may lead to increased susceptibility to infections and other immune-related conditions later in life [22].

Two systematic reviews were undertaken to investigate the correlation between early antibiotic exposure and adverse neonatal outcomes. Esaiassen et al. focused on examining the association with necrotizing enterocolitis, invasive fungal infections, and mortality [10]. Nevertheless, this study was limited by the absence of quantitative data synthesis and the lack of information concerning confounder adjustment, highlighting significant constraints [10]. The other study addressed the risk of necrotizing enterocolitis in preterm infants who underwent extended empirical antibiotic treatment compared to those on a short antibiotic exposure [6]. However, this meta-analysis predominantly utilized unadjusted data rather than adjusted effect sizes, prompting concerns regarding the accuracy of the conclusions drawn. Notably, no study specifically addressed the association between early antibiotic exposure and the subsequent risk of late-onset sepsis in premature infants with sterile cultures. In our investigation, we rely on studies that meticulously adjust for clinical neonatal and maternal confounders. Furthermore, we stratify the analysis based on sample size to mitigate the potential for inflated odds ratios in studies with smaller sample sizes [18].

In this study, prolonged exposure was not significantly associated with LOS (pooled aOR = 1.2, 95% CI 0.99–1.46, *P* = 0.066, PI = 0.66 to 2.19, I = 67%). Limiting the analysis to five studies with sample sizes over 1,000 reduced heterogeneity (I < 0xC6 > = 30%) and provided a more precise confidence interval (pooled aOR = 1.03, 95% CI 0.91–1.15). Notably, the smallest sample sizes in this group, from Kuppala et al. [7] with 305 preterm infants and Shah et al. [8] with 216 preterm infants, yielded the most significant results. Kuppala et al. reported an OR of 2.45 (95% CI 1.28 to 4.67), while Shah et al. reported an OR of 2.1 (95% CI 1.2 to 3.7). This may be attributed to the overestimated odds ratios in studies with small to moderate sample sizes [18]. Likewise, a prior meta-analysis that compared prolonged antibiotic exposure to short-term exposure concerning necrotizing enterocolitis revealed that both the odds ratio and heterogeneity were elevated in studies with smaller sample sizes. Specifically, the analysis indicated a substantial odds ratio of 2.66 (95% CI: 1.54–4.59) with significant heterogeneity (I² = 86%) for studies with sample sizes ranging from 200 to 500. Conversely, studies with sample sizes exceeding 1,000 exhibited a reduced odds ratio of 1.34 (95% CI: 1.16–1.55) and minimal heterogeneity (I² = 9%).

In comparison to preterm infants not exposed to antibiotics, those exposed for a short period exhibited a slightly reduced risk of LOS, with a pooled aOR of 0.87 (95% CI 0.77–0.98, *P* = 0.024). This protective effect could be associated with a milder impact on the gut microbiota during short-term exposure compared to prolonged use, and potentially prompt eradication of suboptimal infections at an early stage. This was supported by a case-control study that found antibiotic administration reduced the risk of LOS in preterm infants. Specifically, the study reported that the risk of LOS was significantly lower with antibiotic use, with an adjusted odds ratio of 0.08 (95% CI: 0.01–0.88; *p* = 0.039). This suggests that administering antibiotics before the onset of sepsis may have a protective effect against developing LOS in preterm infants [14]. In a prior clinical study, the impact of administering antibiotics during the initial 48 h of life on the microbiome of preterm infants was examined in comparison to a control group that did not receive antibiotics [23]. The results of the research indicate that early exposure to antibiotics did not have a detrimental effect on the composition of the microbiome. Noteworthy, there were no notable variances in the overall diversity, richness of species, or beta-diversity of the microbiomes between the preterm infants who were given antibiotics and those who were given a placebo [23]. Moreover, complementary investigations using mouse models revealed no significant distinctions in weight gain, intestinal maturation, or behavior among the offspring colonized with microbiota, thereby reinforcing the outcomes observed in human infants [23]. Similarly, prior studies utilizing preterm pigs as a model demonstrated that prophylactic antibiotics effectively reduced gut bacterial load, enhancing intestinal structure and function, and improved intestinal health by reducing inflammation and enhancing immune function in preterm pigs [15, 24, 25].

### Limitations

In this meta-analysis, we recognize some limitations. The variability in antibiotic exposure duration is one such factor. Although Egger’s test suggests no publication bias, the small number of studies analyzed warrants cautious interpretation. Additionally, despite using multivariable logistic regression to adjust for confounders, there may still be some residual confounding, as certain factors not included in the models could potentially affect the observed associations. Future research should aim to address these limitations by using standardized definitions, improving study quality, and including larger, more diverse sample sizes.

## Conclusion

Our findings indicate that prolonged early antibiotic exposure in preterm infants with sterile cultures does not significantly increase the risk of LOS compared to no antibiotic exposure. Interestingly, a shorter duration of antibiotic exposure might be associated with a slightly lower risk of LOS.

## Electronic supplementary material

Below is the link to the electronic supplementary material.

Supplementary Material 1: The association of antibiotic exposure with late-onset sepsis in preterm infants with sterile culture: a systematic review and meta-analysis studies

## Data Availability

No datasets were generated or analysed during the current study.

## Abbreviations

PRISMA: Preferred Reporting Items for Systematic Reviews and Meta-Analyses
aOR: Adjusted Odds Ratios
PI: Prediction Interval
NICU: Neonatal Intensive Care Unit
NEC: Necrotizing Enterocolitis
LOS: Late-Onset Sepsis
NOS: Newcastle Ottawa Scale
PROM: Premature Rupture of Membranes
GA: Gestational Age
CRIB: Clinical Risk Index for Babies
SGA: Small for Gestational Age
IUGR: Intrauterine Growth Restriction
PIH: Pregnancy-Induced Hypertension
APH: Antepartum Hemorrhage
Apgar: Appearance, Pulse, Grimace, Activity, Respiration
SNAP-II: Score for Neonatal Acute Physiology
SMA: Small for Gestational Age
