# Supplementary material for: The association of early antibiotic exposure with subsequent development of late-onset sepsis in preterm infants: a systematic review and meta-analysis studies
Source: Int J Emerg Med. 2025 Apr 18;18:82. doi: 10.1186/s12245-025-00869-5 (PMC12007201; doi:10.1186/s12245-025-00869-5)
Supplement: Supplementary file 1 — Supplementary Material 1: The association of antibiotic exposure with late-onset sepsis in preterm infants with sterile culture: a systematic review and meta-analysis studies [file 12245_2025_869_MOESM1_ESM.docx]

**Supplementary file

The association of antibiotic exposure with late-onset sepsis in preterm infants with sterile culture: a systematic review and meta-analysis studies**

**Table S1: The search strategy used in this study**

| Search string | Keywords |
| --- | --- |
| #1 | Neonat* OR newborn* OR infant* OR Baby |
| #2 | Antibiotic OR “Anti-Bacterial Agents” OR antimicrobial OR “Antibiotic exposure” OR “prolonged antibiotic exposure” OR “early Antibiotic exposure” |
| #3 | Association OR correlation OR “risk ratio” OR "odds ratio" OR "hazard ratio" |
| #4 | “Late-onset sepsis” OR “Late-onset neonatal sepsis” LOS |
| #5 | #1 AND #2 AND #3 AND #4 |

**Table S2:** Supplementary preferred reporting items for systematic reviews and meta-analyses (PRISMA) checklist.

| **Section and Topic** | **Item #** | **Checklist item** | **Location where item is reported** |
| --- | --- | --- | --- |
| **TITLE** | | |  |
| Title | 1 | Identify the report as a systematic review. | 1 |
| **ABSTRACT** | | |  |
| Abstract | 2 | See the PRISMA 2020 for Abstracts checklist. | 1 |
| **INTRODUCTION** | | |  |
| Rationale | 3 | Describe the rationale for the review in the context of existing knowledge. | 2 |
| Objectives | 4 | Provide an explicit statement of the objective(s) or question(s) the review addresses. | 3 |
| **METHODS** | | |  |
| Eligibility criteria | 5 | Specify the inclusion and exclusion criteria for the review and how studies were grouped for the syntheses. | 4 |
| Information sources | 6 | Specify all databases, registers, websites, organisations, reference lists and other sources searched or consulted to identify studies. Specify the date when each source was last searched or consulted. | 4 |
| Search strategy | 7 | Present the full search strategies for all databases, registers and websites, including any filters and limits used. | 4 |
| Selection process | 8 | Specify the methods used to decide whether a study met the inclusion criteria of the review, including how many reviewers screened each record and each report retrieved, whether they worked independently, and if applicable, details of automation tools used in the process. | 5 |
| Data collection process | 9 | Specify the methods used to collect data from reports, including how many reviewers collected data from each report, whether they worked independently, any processes for obtaining or confirming data from study investigators, and if applicable, details of automation tools used in the process. | 5 |
| Data items | 10a | List and define all outcomes for which data were sought. Specify whether all results that were compatible with each outcome domain in each study were sought (e.g. for all measures, time points, analyses), and if not, the methods used to decide which results to collect. | 5,6 |
|  | 10b | List and define all other variables for which data were sought (e.g. participant and intervention characteristics, funding sources). Describe any assumptions made about any missing or unclear information. | 4,16 |
| Study risk of bias assessment | 11 | Specify the methods used to assess risk of bias in the included studies, including details of the tool(s) used, how many reviewers assessed each study and whether they worked independently, and if applicable, details of automation tools used in the process. | 6 |
| Effect measures | 12 | Specify for each outcome the effect measure(s) (e.g. risk ratio, mean difference) used in the synthesis or presentation of results. | 6 |
| Synthesis methods | 13a | Describe the processes used to decide which studies were eligible for each synthesis (e.g. tabulating the study intervention characteristics and comparing against the planned groups for each synthesis (item #5)). | 6,7 |
|  | 13b | Describe any methods required to prepare the data for presentation or synthesis, such as handling of missing summary statistics, or data conversions. | 6 |
|  | 13c | Describe any methods used to tabulate or visually display results of individual studies and syntheses. | 8,9,10 |
|  | 13d | Describe any methods used to synthesize results and provide a rationale for the choice(s). If meta-analysis was performed, describe the model(s), method(s) to identify the presence and extent of statistical heterogeneity, and software package(s) used. | 6 |
|  | 13e | Describe any methods used to explore possible causes of heterogeneity among study results (e.g. subgroup analysis, meta-regression). | 6 |
|  | 13f | Describe any sensitivity analyses conducted to assess robustness of the synthesized results. | 6,7 |
| Reporting bias assessment | 14 | Describe any methods used to assess risk of bias due to missing results in a synthesis (arising from reporting biases). | 6 |
| Certainty assessment | 15 | Describe any methods used to assess certainty (or confidence) in the body of evidence for an outcome. | 6 |
| **RESULTS** | | |  |
| Study selection | 16a | Describe the results of the search and selection process, from the number of records identified in the search to the number of studies included in the review, ideally using a flow diagram. | 7 |
|  | 16b | Cite studies that might appear to meet the inclusion criteria, but which were excluded, and explain why they were excluded. | 8-11 |
| Study characteristics | 17 | Cite each included study and present its characteristics. | 8-11 |
| Risk of bias in studies | 18 | Present assessments of risk of bias for each included study. | 11,12 |
| Results of individual studies | 19 | For all outcomes, present, for each study: (a) summary statistics for each group (where appropriate) and (b) an effect estimates and its precision (e.g. confidence/credible interval), ideally using structured tables or plots. | 11,12 |
| Results of syntheses | 20a | For each synthesis, briefly summarise the characteristics and risk of bias among contributing studies. | 11,12 |
|  | 20b | Present results of all statistical syntheses conducted. If meta-analysis was done, present for each the summary estimate and its precision (e.g. confidence/credible interval) and measures of statistical heterogeneity. If comparing groups, describe the direction of the effect. | 11,12 |
|  | 20c | Present results of all investigations of possible causes of heterogeneity among study results. | 11 |
|  | 20d | Present results of all sensitivity analyses conducted to assess the robustness of the synthesized results. | 11 |
| Reporting biases | 21 | Present assessments of risk of bias due to missing results (arising from reporting biases) for each synthesis assessed. | 11 |
| Certainty of evidence | 22 | Present assessments of certainty (or confidence) in the body of evidence for each outcome assessed. | 11 |
| **DISCUSSION** | | |  |
| Discussion | 23a | Provide a general interpretation of the results in the context of other evidence. | 12-15 |
|  | 23b | Discuss any limitations of the evidence included in the review. | 15 |
|  | 23c | Discuss any limitations of the review processes used. | 15 |
|  | 23d | Discuss implications of the results for practice, policy, and future research. | 15 |
| **OTHER INFORMATION** | | |  |
| Registration and protocol | 24a | Provide registration information for the review, including register name and registration number, or state that the review was not registered. | N/A |
|  | 24b | Indicate where the review protocol can be accessed, or state that a protocol was not prepared. | N/A |
|  | 24c | Describe and explain any amendments to information provided at registration or in the protocol. | N/A |
| Support | 25 | Describe sources of financial or non-financial support for the review, and the role of the funders or sponsors in the review. | 16 |
| Competing interests | 26 | Declare any competing interests of review authors. | 16 |
| Availability of data, code and other materials | 27 | Report which of the following are publicly available and where they can be found: template data collection forms; data extracted from included studies; data used for all analyses; analytic code; any other materials used in the review. | N/A |

**Table S3: NEWCASTLE - OTTAWA QUALITY ASSESSMENT SCALE** **COHORT STUDIES**

| Last name of the first  author (publication year) | **Selection** | | | | **Comparability** | | | | **Outcome** |
| --- | --- | --- | --- | --- | --- | --- | --- | --- | --- |
|  | Representativeness of  the exposed cohort | Selection of of  the non- exposed cohort | Ascertainment of exposure | 4Demonstration that outcome of  interest was not present at start of study | Comparability of cohorts based  on the design or analysis | Assessment  of outcome | Was follow-up long enough  for outcomes to occur | Adequacy of follow-up  of cohorts |  |
| Kuppala (2011)[7] | * |  | ** | * | ** |  | * | * | * |
| Cotten (2009)[3] | * |  | ** | * | ** |  | * | * | * |
| Ting (2019)[13] | * |  | ** | * | ** |  | * | * | * |
| Greenberg (2019)   [20] | * |  | ** | * | ** |  | * | * | * |
| Dierikx (2022)[12] | * |  | ** | * | ** |  | * | * | * |
| Fajardoa (2018) [9] | * |  | ** | * | ** |  | * | * | * |
| Yu (2023) [21] | * |  | ** | * | ** |  | * | * | * |
| Vatne (2023)[11] | * |  | ** | * | ** |  | * | * | * |
| Shah (2013)[8] | * |  | ** | * | ** |  | * | * | * |

**Table S4: Cohort Demographics and Designs of the included studies**

| Author | Setting/Country | Study Design | Birth Years of Cohort | Total Participants | Total number of infants with **prolonged** early exposure to antibiotics | Total number of infants with **short** early exposure to antibiotics. | Total number of infants **without** exposure to antibiotics  **(0 days)** | Late-Onset Sepsis Cases by Duration of Exposure |
| --- | --- | --- | --- | --- | --- | --- | --- | --- |
| Kuppala[7] | USA (Three NICUs, Cincinnati, Ohio) | Retrospective cohort | April 2000 – December 2004 | 365 | 130 (≥5 days) | 175 (1–4 Days) | 60 | 0 Days 7 (11.7%),  1–4 Days: 23 (13.1%),  ≥5 Days: 46 (35.4%) |
| Shah[8] | Australia (Perth, Western Australia) | Retrospective cohort | January 2009 – December 2010 | 216 | 120  (≥4 days) | 96   (<4 days) | NA | <4 days: 18 (20.7%),  ≥4 days: 69(79.3%) |
| Ting[13] | Canada (29 NICUs, Canadian Neonatal Network) | Retrospective cohort | January 2010 – December 2016 | 14,207 | 5,856  (≥4 days) | 5,401  (<4 days) | 2,950 | 0 Days: 258 (9%), <4 Days: 666 (12%), 4–7 Days: 1,149 (20%) |
| Cotten[3] | USA (NICHD Neonatal Research Network, 19 centers) | Retrospective cohort | September 1998 – December 2001 | 5,693 | 2,147 (≥5 days) | 1,892  (<5 days) | NA | ≥5 days of initial empirical antibiotic treatment associated with increased LOS (aOR: 1.09, 95% CI: 0.95–1.26) **^α^** |
| Greenberg[20] | USA (NICHD Neonatal Research Network, 13 centers) | Retrospective cohort | January 2008 – December 2014 | 5,730 | 2,526 (≥5 days) | 3,204 | NA | < 5 days:670   ≥5 days: 540 |
| Dierikx[12] | Netherlands & Belgium (9 NICUs) | Prospective cohort | October 2014 – January 2019 | 1,259 | 320 (≥3 days) | 802 (≤3 days) | 137 | 0 days: 45 (32.8%) ≤3 days: 266 (33.2%) >3 days: 110 (34.4%), |
| Fajardo[9] | Canada (Foothills Medical Centre NICU) | Retrospective cohort | January 2010 – December 2013 | 620 | 238 (>5 days) | 382 (≤5 days) | NA | ≤5 Days: 35 (9.1%),  >5 Days Antibiotics: 59 (24.7%) |
| Yu[21] | China (25 tertiary NICUs) | Prospective cohort | May 2015 – April 2018 | 21,540 | 14,702 (5–7 days) | 3,600  (1–4 days) | 3,238  (0 days) | 0 days 303 (9.4%) 1-4 days: 295 (8.2%) 5–7 days 1556 (10.6%) vs. |
| Alsafadi [19] | Saudi Arabia (Three NICUs) | Retrospective cohort | January 2013 – March 2017 | 587 | Not specified | Not specified | NA | Total Proven LOS Cases: 85 neonates (14.4% of total participants  ≥4 days increased LOS risk (aOR: 1.13, 95% CI: 1.027–1.25) **^α^** |
| NA: Not applicable (the study focuses solely on comparing infants with prolonged versus short antibiotic exposure).  aOR: Adjusted odds ratio.  α: The study reports adjusted odds ratios directly without specifying exact numerical values | | | | | | | | |
